# Bi-allelic intermediate ATXN2 repeat expansions are associated with slow progressing, leg-onset familial ALS

**DOI:** 10.1136/bmjno-2025-001417

**Published:** 2026-02-18

**Authors:** Koen Cedric Demaegd, Wouter Koole, Joke JFA van Vugt, Jan Willem Dankbaar, Jeroen Hendrikse, A Nazlı Başak, Mamede de Carvalho, Philippe Corcia, Philippe Codron, Emilien Bernard, Claire Guissart, Philippe Couratier, Mónica Povedano Panades, Pieter A van Doorn, Bart P Warrenburg, Johnathan Cooper-Knock, ALS Sequencing Consortium, Project MinE PI’s, Patrick Vourc’h, R Jeroen Pasterkamp, Wouter van Rheenen, Philip van Damme, Leonard H van den Berg, Jan Herman Veldink, Michael A van Es

**Affiliations:** 1 Department of Neurology, UMC Utrecht, Utrecht, the Netherlands; 2 Laboratory of Neurobiology, Department of Neuroscience, KU Leuven, Leuven, Belgium; 3 Department of Genetics, UMC Utrecht, Utrecht, Utrecht, the Netherlands; 4 Department of Radiology, UMC Utrecht, Utrecht, the Netherlands; 5 Suna and İnan Kıraç Foundation, Neurodegeneration Research Laboratory (NDAL), KUTTAM, Koç University School of Medicine, Istanbul, Turkey; 6 University of Lisbon, Lisbon, Lisbon, Portugal; 7 Universite de Tours Faculte de Medecine, Tours, Centre-Val de Loire, France; 8 Angers University Hospital, Angers, Pays de la Loire, France; 9 Rare Disease Reference Center for ALS (CRMR SLA), Pierre Wertheimer Hospital, Hospices Civils de Lyon, Institut NeuroMyoGène-PGNM, Claude Bernard University, Lyon, France; 10 Montpellier University, Montpellier, Occitanie, France; 11 ALS Centre, Departement of Neurology, CHU Limoges, Limoges, Aquitaine-Limousin-Poitou-Charentes, France; 12 Department of Neurology, Hospital Universitari de Bellvitge, L’Hospitalet de Llobregat, Catalunya, Spain; 13 Department of Neurology, Erasmus MC, Rotterdam, the Netherlands; 14 Department of Neurology, Radboudumc, Nijmegen, the Netherlands; 15 Department of Neurology, Sheffield Institute for Translational Neuroscience (SITraN), Sheffield, South Yorkshire, UK; 16 Department of Translational Neuroscience, UMC Utrecht, Utrecht, Utrecht, the Netherlands; 17 Department of Neurology, University Hospitals Leuven, Leuven, Belgium

**Keywords:** ALS, GENETICS, CLINICAL NEUROLOGY

## Abstract

**Objectives:**

The identification of bi-allelic intermediate *ATXN2* repeat expansions in a pedigree with amyotrophic lateral sclerosis (ALS) through clinical testing prompted us to investigate its relevance in the wider ALS population.

**Methods:**

*ATXN2* repeat size was assessed in a large international cohort of ALS patients (n=6653 from Project MinE) and in neurologically intact control populations (n=13 515 controls from Project MinE and gnomad). For bi-allelic cases, we retrieved medical records, family history and MRI imaging. For familial cases, we obtained DNA samples from relatives for segregation analyses.

**Results:**

In total, we identified bi-allelic intermediate *ATXN2* repeat expansions in five familial cases from three different pedigrees and five apparently sporadic cases. There is a relatively homogeneous phenotype characterised by lower limb onset and long survival (median 6 years) without significant cerebellar atrophy. Bi-allelic expansions were absent in controls (0 out of 13 515).

**Discussion:**

Here we report an apparently novel autosomal recessive form of familial ALS caused by bi-allelic intermediate *ATXN2* repeat expansions, which is characterised by high penetrance, lower limb onset and slow progression. Although rare, testing for *ATXN2* expansions should be performed in the clinical setting given its relevance to prognosis and genetic counselling.

**WHAT IS ALREADY KNOWN ON THIS TOPIC**

- Heterozygous intermediate CAG repeat expansions in the *ATXN2* gene are a risk factor for amyotrophic lateral sclerosis (ALS), while repeats of 34 and longer (full length) cause autosomal dominant spinocerebellar ataxia type 2. The association between bi-allelic intermediate *ATXN2* repeat expansions and ALS is unknown.

**WHAT THIS STUDY ADDS**

- This study identifies a previously unrecognised autosomal recessive form of ALS caused by bi-allelic intermediate *ATXN2* repeat expansions. It establishes that these expansions are absent in controls and lead to a distinctive ALS phenotype with lower limb onset, slow disease progression and long survival.

**HOW THIS STUDY MIGHT AFFECT RESEARCH, PRACTICE OR POLICY**

- Recognising this inheritance pattern informs prognosis and supports routine *ATXN2* testing in ALS diagnostics and counselling.

## Introduction

 The *ATXN2* gene contains a repeated CAG sequence encoding a polyglutamine tract within the ataxin-2 protein. The length of these CAG repeats varies among individuals and may become expanded. Up to 28 CAG repeats are common in healthy individuals. CAG repeats between 29 and 33 (intermediate repeats) are rare in control populations (<0.4%) but have been identified at higher frequencies in cohorts of patients with parkinsonism and amyotrophic lateral sclerosis (ALS). Intermediate repeats are therefore considered to be risk factors for ALS and parkinsonism.^1^,^4^

The OR for ALS susceptibility increases with *ATXN2* repeat size; 1.68 for 29 to 8.37 for 32 repeats.^5^ Repeats of 34 and longer (full length) cause autosomal dominant spinocerebellar ataxia type 2 (SCA2). CAG repeats have been shown to be genetically unstable, meaning that the size of repeat may increase. This leads to longer CAG repeats and more severe disease and younger onset in subsequent generations, known as anticipation.^6^

Importantly, increased risk for ALS and parkinsonism is associated with heterozygous intermediate expansions, and SCA2 is an autosomal dominant disorder caused by full length expansions. Through previous work and through genetic testing in a clinical setting, we discovered bi-allelic *ATXN2* intermediate expansions in patients with ALS.^7^ These unusual findings prompted us to further investigate the relevance of bi-allelic *ATXN2* expansions to ALS at a larger scale. As a result, we describe a cohort of ten patients with a nearly fully penetrant disease and a relatively uniform phenotype.

## Methods

We analysed whole-genome sequencing data from an international ALS genetics consortium, Project MinE, consisting of 6653 cases.^8^ Based on data from a large-scale meta-analysis, we defined 29–34 repeats as intermediate expansions.^5 7^ The frequency of bi-allelic *ATXN2* repeats was also assessed in control populations without neurological disease, for which we used gnomAD and Project MinE controls (n=13 515). For all bi-allelic *ATXN2* cases, we subsequently retrieved medical records and family history. For familial cases, we obtained DNA samples from family members for segregation analyses. In family members, we assessed *ATXN2* repeat length using repeat primed PCR. The phenotype was studied using medical records from which we derived age and site of onset, survival, etc and MRI imaging if available. Cerebellar MRI images were assessed by two experienced neuroradiologists, blinded for phenotype.

## Results

We identified a total of 10 cases with bi-allelic expansions, five of which were familial (stemming from three pedigrees) and five were apparently sporadic cases. Bi-allelic expansions were not seen in the non-neuro controls from Project MinE or the gnomAD database (0 out of 13 515 individuals). Additionally, we conducted a literature review, which also did not identify any reports of bi-allelic *ATXN2* expansions in healthy controls (see online supplemental material).

Heterozygous repeats between 29 and 34 were seen in 0.97% of healthy controls and in 2.99% of ALS patients. The expected frequency of bi-allelic intermediate repeat expansion carriers in healthy controls is 0.0097*0.0097=0.00009 and adds further evidence that the bi-allelic intermediate repeat expansions in multiple ALS cases are not a chance finding.

**Figure 1:**
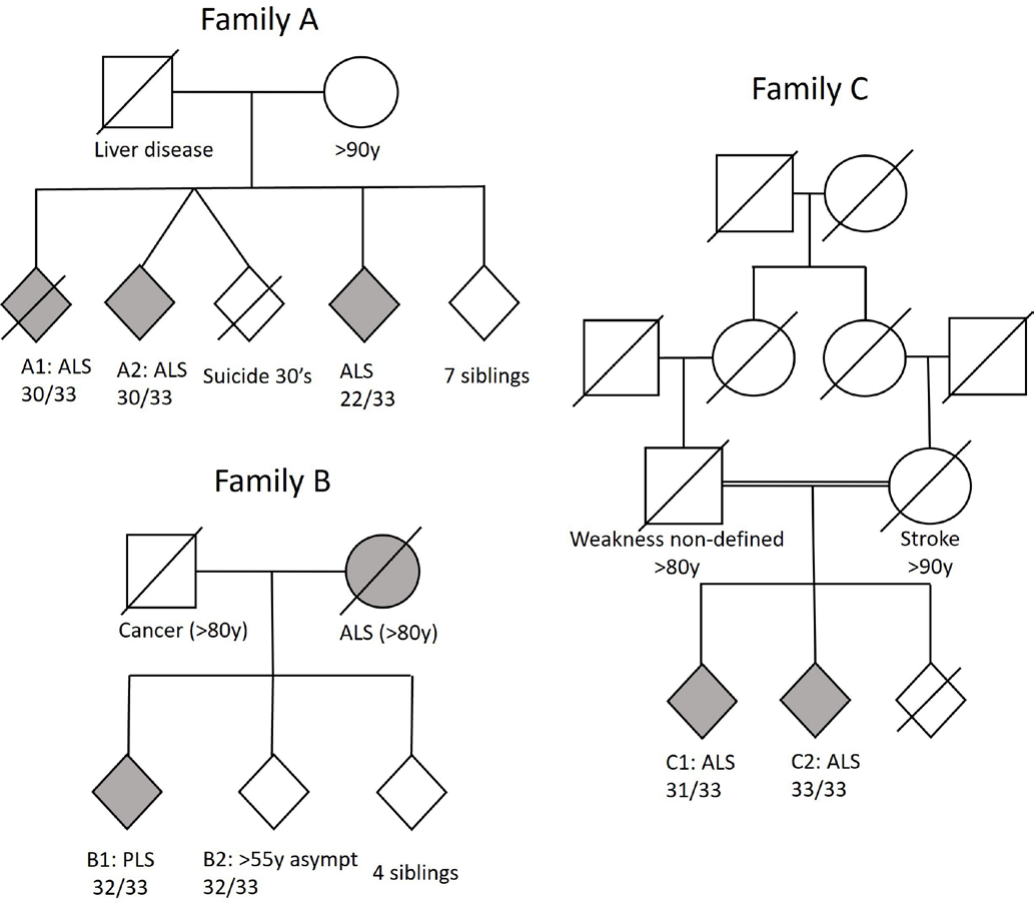
Pedigrees of familial cases with bi-allelic *ATXN2*-associated ALS. ALS, amyotrophic lateral sclerosis. PLS, primary lateral sclerosis

A brief description of the three pedigrees (figure 1) is provided below and a summary of clinical findings for all cases is provided in table 1. Genetic testing did not reveal clinically relevant variants in other *FALS* genes in any of the pedigrees or sporadic cases. Unfortunately, patients did not undergo a standardised neuropsychological screening. However, based on the medical records, there does not appear to be a clear cognitive or behavioural phenotype. None of the family members of our cases had Parkinson’s disease or Frontotemporal dementia. Cerebellar involvement was not apparent on neurological examination (ataxia, nystagmus, etc) or on MRI.

**Table 1:** Clinical information of all bi-allelic *ATXN2* motor neurone disease cases

Case	Phenotype	Repeats	Onset age	Diagnostic delay (months)	ΔFRS	Survival (years)	Region
Family A-1	ALS	30/33	65	20	0.5	5.1	Leg
Family A-2	ALS	30/33	60	54	0.05	>6	Spinal
Family B-1	PLS	32/33	62	95	0.1	>10	Leg
Family B-2	Asympt	32/33	NA	NA	NA	NA	NA
Family C-1	ALS	31/33	73	51	0.05	20	Leg
Family C-2	ALS	33/33	72	46	0.07	>20	Leg
Sporadic 1	ALS	29/31	52	16	0.16	>7	Leg
Sporadic 2	ALS	30/30	76	18	Unknown	3.5	Leg
Sporadic 3	ALS	31/31	52	11	Unknown	>10	Leg
Sporadic 4	ALS	30/30	58	10	0.83	6	Leg
Sporadic 5	ALS	30/31	78	2	8.1	0.6	Leg

ALS, amyotrophic lateral sclerosis; PLS, primary lateral sclerosis.

Family A was a non-consanguineous pedigree from France that consisted of 11 siblings of whom three were diagnosed with ALS. Patient A-1 first noticed fasciculations and weakness in the legs in his mid-60s. The patient was diagnosed with ALS 1.5 years after onset and had slow progression of weakness. The patient died a few years later from respiratory failure.

Patient A-2 was initially diagnosed with cramp fasciculation syndrome as there were no upper motor neuron signs, weakness or abnormalities on needle-electromyography(EMG). Four years later, he developed dysarthria, pseudobulbar affect and weakness of the left arm leading to a revised diagnosis of ALS. In hindsight, under the Miami framework, one would probably reclassify the cramps and fasciculations as mild motor impairment, representing the earliest manifestations of the disease.

The third affected sibling in this pedigree was heterozygous for the *ATXN2* intermediate repeat (22/33). Carriership of one intermediate *ATXN2* repeat also confers an increased risk to develop ALS.^5^

Family B was a pedigree from the Netherlands without evidence of consanguinity. The index case (Patient B1) presented in his early 60s with difficulty walking. On examination, spasticity and hyperreflexia of the left leg as well as hyperreflexia in both arms and pseudobulbar reflexes were found without weakness, atrophy or fasciculations. Needle EMG, however, demonstrated loss of lower motor neurons in both legs and right hand. MRI showed slight atrophy of the brain stem, cerebellum and bitemporal regions. Three out of five siblings were tested for *ATXN2* repeats, of which one also carried a bi-allelic repeat (32/33). This individual is asymptomatic at 55 years - approximately a decade younger than the average age of onset of other bi-allelic carriers – and under clinical surveillance, as his condition could evolve.

Family C was a consanguineous pedigree from the Netherlands,^7^ in which two siblings had both been diagnosed with ALS. Patient C-1 presented at an age over 75 with slowly progressive difficulty walking and climbing stairs for 4 years.

The sibling (patient C-2) presented in his mid-70s and had also been having trouble walking for about 4 years. Neurological examination showed a spastic gait as well as dysarthria, pseudobulbar affect, drooling, hyperreflexia, fasciculations and hypertonia in all limbs. Weakness was most pronounced in the legs, tongue and neck flexors. Despite some atypical sensory deficits, extensive ancillary investigations in both cases did not yield evidence for any diagnosis other than ALS.

One sibling is still alive with survival >20 years to date. The unaffected third sibling had a heterozygous *ATXN2* repeat (22/33) and died from an unrelated cause in his 80s. The father also suffered from a progressive gait disorder with muscle weakness that appears to have spread proximally and to the bulbar region over a period of 10 years. He never sought medical advice and died in his early 80s.

### Apparently, sporadic cases

Apart from the aforementioned cases with clear familial inheritance, we identified five more patients who have no clear family history of motor neurone disease (MND) based on the available records. See table 1 for details.

### Genotype-phenotype correlation

In total, we describe eleven carriers of intermediate *ATXN2* repeats on both alleles from European descent, of which ten MND patients and one asymptomatic individual well below the average age of onset. The average age of onset is 64 years. The nearly homogenous phenotype of spinal onset (9 out of 10 lower limb) and long survival (median 6 years, IQR (4.6 to 8.5) years) is striking.

## Discussion

In this study, we present for the first time a case series of MND patients with bi-allelic *ATXN2* intermediate repeat expansions. Cases show a uniform phenotype characterised by motor neuron disease with spinal onset (predominantly lower limb), slow progression and long survival without ataxia or overt cognitive or behavioural deficits.

The absence of bi-allelic intermediate repeats in over 13 000 healthy controls, combined with the observation that all bi-allelic repeat carriers (except one, significantly younger than the typical onset age) developed MND, suggests that bi-allelic carriership could be pathogenic.

Indeed, the observed pattern of inheritance in our pedigrees is compatible with autosomal recessive transmission. Nevertheless, additional pedigrees, larger data sets and functional data are required to definitively demonstrate pathogenicity of bi-allelic repeats.

In summary, our findings suggest that bi-allelic intermediate *ATXN2* repeats cause a novel form of highly penetrant ALS associated with lower limb onset and long survival. Clearly, bi-allelic *ATXN2* repeats are rare, but should nonetheless be tested in the clinical setting given its relevance to both prognosis and genetic counselling. Further studies are required to elucidate the underlying pathophysiology and develop targeted therapies.

## Supplementary material

10.1136/bmjno-2025-001417 online supplemental file 1

## Data Availability

Data are available upon reasonable request.

## References

[1] Demaegd KC, Kernan A, Cooper-Knock J (2025). An observational study of pleiotropy and penetrance of amyotrophic lateral sclerosis associated with CAG-repeat expansion of ATXN2. Eur J Hum Genet.

[2] Elden AC, Kim H-J, Hart MP (2010). Ataxin-2 intermediate-length polyglutamine expansions are associated with increased risk for ALS. Nature New Biol.

[3] Furtado S, Payami H, Lockhart PJ (2004). Profile of families with parkinsonism-predominant spinocerebellar ataxia type 2 (SCA2). Mov Disord.

[4] Vieira de Sá R, Sudria-Lopez E, Cañizares Luna M (2024). ATAXIN-2 intermediate-length polyglutamine expansions elicit ALS-associated metabolic and immune phenotypes. Nat Commun.

[5] Sproviero W, Shatunov A, Stahl D (2017). ATXN2 trinucleotide repeat length correlates with risk of ALS. Neurobiol Aging.

[6] Sena LS, Dos Santos Pinheiro J, Hasan A (2021). Spinocerebellar ataxia type 2 from an evolutionary perspective: Systematic review and meta-analysis. Clin Genet.

[7] Van Damme P, Veldink JH, van Blitterswijk M (2011). Expanded ATXN2 CAG repeat size in ALS identifies genetic overlap between ALS and SCA2. Neurology (ECronicon).

[8] Project MinE ALS Sequencing Consortium (2018). Project MinE: study design and pilot analyses of a large-scale whole-genome sequencing study in amyotrophic lateral sclerosis. Eur J Hum Genet.

