# Supplementary material for: Bi-allelic intermediate ATXN2 repeat expansions are associated with slow progressing, leg-onset familial ALS
Source: BMJ Neurol Open. 2026 Feb 18;8(1):e001417. doi: 10.1136/bmjno-2025-001417 (PMC12918675; doi:10.1136/bmjno-2025-001417)
Supplement: online supplemental file 1 [file bmjno-8-1-s001.docx]

**Supplement – Methods**

*Informed consent of all cases was obtained and approval of the protocol obtained by the University Medical Center Utrecht Medical Ethics Committee, Utrecht. ExpansionHunter v5.0.0 was used to determine the ATXN2 repeat sizes from Project MinE genomes^1, 2^. Out of* *6,992 Project MinE genomes sequenced 150bp paired-end with HiSeqX, of which 5,242 MND cases and 1,750 healthy controls, 5 bi-allelic repeat carriers from four countries were retrieved. Local PIs were contacted for clinical information, leading among others to family A in which another case was found. In four cases the WGS result was confirmed with PCR. PCR was performed with forward primer 5’-[6FAM]GGGCCCCTCACCATGTCG-3’ and reverse primer 5’– CGGGCTTGCGGACATTGG – 3’ and PCR cycles: 1 min at 96 C, 35 cycles (30 sec at 96 C, 45 sec at 62 C, 1 min at 72 C), and 5 min at 72 C. ATXN2 repeat lengths were determined from PCR products by capillary electrophoresis and GeneMarker software (SoftGenetics). In one case, CAG trinucleotide repeats expansions in the ATXN2 gene were examined by fragment-length analysis using the following primers: 5’-TTCCGGCGTCTCCTTGGC (forward primer) and 5’-GACGAGGACGGCGAAGGC-3’ (reverse primer). The forward primer was marked with 6-FAM fluorophore. The number of CAG repeats were determined using capillary electrophoresis.*

*Out of* *2,087 Project MinE genomes, sequenced 100 bp paired-end with HiSeq2000 , of which 1,411 MND cases and 676 controls, no individual was found with both ATXN2 alleles >= 29.*

*For our literature search, Pubmed dd August 30th, 2023, was searched on "ATXN2 bi-allelic", "Ataxin 2 bi-allelic", "SCA2 bi-allelic", "ATXN2 homozygous", "SCA2 homozygous", "ataxin 2 homozygous", "homozygous expanded trinucleotide repeat ALS", "homozygous expanded trinucleotide repeat ATXN2", "homozygous expanded trinucleotide repeat SCA2".*

*The ATXN2 Short Tandem Repeat genotypes of 11,089 PCR-free gnomAD genomes (v3.1.3) without neurodegenerative diseases were kindly provided by Ben Weisburd (Broad Institute of MIT and Harvard, Cambridge, Massachusetts) ^3^. None of these genomes had >= 29 ATXN2 repeats on both alleles.*

**References**

1. Dolzhenko E, Deshpande V, Schlesinger F, et al. ExpansionHunter: a sequence-graph-based tool to analyze variation in short tandem repeat regions. Bioinformatics 2019;35:4754-4756.

2. Dolzhenko E, van Vugt J, Shaw RJ, et al. Detection of long repeat expansions from PCR-free whole-genome sequence data. Genome Res 2017;27:1895-1903.

3. Ben Weisburd GV, Nick Watts. The Addition of Short Tandem Repeat Calls to gnomAD (v3.1.3) [online]. Available at: <https://gnomad.broadinstitute.org/news/2022-01-the-addition-of-short-tandem-repeat-calls-to-gnomad/>. Accessed August 16, 2024.
